# Participation and significance of self-help groups for social development: exploring the community capacity in Ethiopia

**DOI:** 10.1186/2193-1801-3-189

**Published:** 2014-04-15

**Authors:** Elias Teshome, Mulumebet Zenebe, Henok Metaferia, Sibhatu Biadgilign

**Affiliations:** Center for Gender Studies, Addis Ababa University, Addis Ababa, Ethiopia; School of Public Health, Addis Ababa University, Addis Ababa, Ethiopia; Department of Epidemiology and Biostatistics, College of Public Health and Medical Science, Jimma University, Jimma, Ethiopia

## Abstract

**Background:**

There are various Civil Society Organizations (CSOs) in Ethiopia among which the ‘Idir’ is a social and financial institution widespread both in urban and rural areas of the country. So the objectives of this study is to investigate how women members perceive the contribution of iddirs toward improving their lives and to determine whether and to what extent participation in iddirs has social impacts on their lives.

**Methods:**

A cross-sectional qualitative study using Key Informant In-Depth Interviews (KII) and Focus Group Discussions (FGDs) was conducted in Addis Ababa, Addis Ketema Sub-city. Ethiopia. Data was collected using a semi-structured interview questionnaire and FGD guideline. Analysis of the data was made manually using thematic framework analysis method.

**Result:**

Though their iddir doesn’t provide financial assistance, all the participants revealed the importance of installing credit mechanisms in their iddirs. However, they mentioned the inability of their respective iddirs in assisting members with their financial needs. One major difficulty mentioned was lack of capital. The participants demonstrated that the contribution of iddir in their well-being was more indispensable than the contributions of other voluntary associations they are acquainted with, such as iqub and mahiber. Especially iddir was regarded as crucial and unique in meeting emotional needs. As well, iddirs’ meetings are ideal places where women share experience; discuss issues of pressing concern and their worries. Other benefit of iddir include opportunities for social interaction, risk sharing and development of friendships, dispute resolution, Sharing and using timely information more effectively, Lower level of funeral services anxiety, Improvement of self confidence and leadership role, reciprocity and coexistence and trust.

**Conclusion:**

Women’s *iddirs* are the viable basis in the creation of social network which plays crucial roles in providing solutions to social and economic challenges women are facing. There was a general consensus by the participants that their *iddirs* were unable to offer financial assistances. Enabling women’s *iddirs* to be independent of borrowing from banks is also indispensable and trainings on effective use of credits and the positive role of social capital formed in women’s *iddirs* is relevant.

## Introduction

The role of civil society organizations in mobilizing the community to participate in developmental activities has been argued to have comparative advantages as compared to other approaches such as state intervention in many ways (Rooy 1998). In the past, development policies and strategies of developing countries have been dominated by, either private sector led market or state led development strategy for health, economic growth and poverty eradication. However, third world countries like Ethiopia generally lack such modern organizations, which can provide the necessary services to the beneficiaries at the local level (Aredo and Asefa 1998). ‘Iqqub’ and ‘Idir’ are among the most important financial institutions in Ethiopia. The iddir is an informal financial and social institution that is almost ubiquitous throughout (Aredo 1993). According to Sable (1986) the original purpose of iddir was the burial of the dead. Today, iddir provides a much wider range of services including financial and material assistance and consolations for a member in the event of difficulties as well as entertainment as the case may be (Sable 1986).

The expansion of the iddir in urban areas is perhaps associated with growing social insecurity. It has been observed that in Addis Ababa the iddir has been growing at a fast rate. According to Sable (1986) out of total residents in Addis Ababa, more than half households were members of iddirs (Sable 1986). The functions of iddirs are not limited to the provision of insurance and psychological support for members; iddirs are often involved in community development programmes such as in construction of roads and schools, as well as installation of public utilities (Aleme 1973;Mekuria 1973). Other literature focuses on development initiatives using community-based organizations. One strand is related to health insurance, since many initiatives have developed around voluntary but community-based health insurance (CBHI) schemes (Atim 1998;Jutting 2003). Discussion on membership based indigenous insurance associations in existing literature is very limited at present (Dercon et al. 2006). In the Ethiopian context, Aredo has discussed funeral associations in some detail from an economic point of view (Aredo 1993,1998).

Due to government and market failure in poverty alleviation and sustainable development, particularly in developing countries, the civil society organizations (CSOs) have long been identified as a third category of actors in development (Sitz 1995).

In Ethiopia, varieties of such CSOs exist among which Iddirs are the most widespread, institutions in both urban and rural areas primarily established to provide mutual aids in burial matters at times of death but also address other community concerns at times (Pankhurst 1998). Similarly, Yan (2004) explained that mutual help is essential to the human race to progress to a full humanity. Only through a reciprocal linkage among people would the progress of the whole human race be possible (Yan 2004). Furthermore, studies have demonstrated that participation in voluntary associations have multiple benefits (Bown (2000). In the same way, Beck and Eichler (2000) emphasized that consensus organizing involves a strategic leadership-building process that develops empowerment and stimulates fresh thinking and action on a range of issues related to poverty (Beck and Eichler 2000). Moreover, Breton (2001) stated that neighbor networks make a bigger contribution to resilience in socio-economically deprived neighborhoods (Breton 2001).

So far there is a paucity of documented evidence that clearly shows the contribution and implementation of *iddir* to society and the country at large. Therefore, the objectives of this study is to investigate how women members perceive the contribution of iddirs toward improving their lives and to determine whether and to what extent participation in *iddirs* has social impacts (strengthening social networks, mutual trust, solidarity, information sharing, etc.) on their lives.

## Methods

### Study settings and context

Addis Ababa is divided for administrative purposes into sub-cities, and each sub-city is further divided into *kebeles*. This study was carried out in Addis Ketema Sub-city, which is one of the ten sub-cities with a population of 320,389 who reside in 9 *kebeles* (Addis Ababa City Administration 2007). Several different ethnic groups reside in the area, including *Amhara*, *Oromo*, *Tigrie* and *Gurage*.

### Study design and participants

The study design was a cross-sectional with data collected primarily by qualitative data collection methods-Focus Group Discussion (FGD) and in-depth interview (IDI). The target population was all women’s *iddirs* members in the sub-city. But the actual population from which the survey data were collected was members of five women’s *iddirs’* in *kebele* 10.

### Data collection instruments

The field work began in the second week of April 2008 and completed in the third week of April 2008. The information relevant for the study was collected by a combination of different instruments. This is based on those that had been successfully used by the researchers in previous studies on self-help associations.

### In-depth interview

Seeing that the aim of this paper is to investigate the roles of *iddirs* in promoting women’s social and economic well-being as perceived by the women themselves, their personal experiences were very fundamental. In-depth interviews were held with six members and four *iddirs’* leaders (one of them was a retired leader) by virtue of their leadership positions. The interviews consisted of both open and closed-ended questions based on interview guides used as checklist on the topics to be discussed. The questions had to be prepared into the context in which they were used. Thus, different terminologies were adapted into a language which was proper for women who have had little or no education. Interview was also supported by writing field notes and each interview lasted between 45 and 55 minutes.

### Focus group discussion

Individual interviews with the selected members and leaders were supplemented by group discussions with key participants. Accordingly, three focus group discussions (n = 8 members for each FGD) were held with selected *iddirs’* members. Efforts were made to conduct FGD with three age groups, namely relatively young members (24–35 years of age), middle aged members (36–47 years of age) and the elderly (≥ 48 years of age) to insure that all age groups were included in the study. The two trained individuals were assigned to take notes, and the participants’ exact words were used in the paper to give a firsthand representation to the women’s emotions and experiences. In all the discussions the researcher was a facilitator, and was able to keep the discussion on track and make sure that every participant was heard.

### Data analysis

Preliminary manual analysis was an inherent part of the data collection. Contact summaries were written in English for each interview. The contact summary summarizes the output of each contact with respect to the themes that was formulated initially, summarizes the general output of the encounter, indicate other relevant issues that come up during the contact and finally it indicates the issues that are already saturated and those that need further clarification. The analysis primarily focuses on textual data in the form of expanded field notes and transcripts of recorded interviews. The process of analysis started by reading and by rereading the texts and notes. Attempts were made to list the themes identified. The data found from the focus group discussions and the in-depth interviews were categorized into themes and presented descriptively. Finally an overall interpretation emerged showing how thematic areas relate to one another.

### Ethical considerations

Ethical clearance was obtained from institutional review board (IRB) of Addis Ababa University. Permission letters were also obtained from different concerned authorities and offices of Addis Ababa. Verbal consent was also obtained from each individual respondent during data collection after a through explanation of the purpose of the study. Participants were well informed about issues such as anonymity of responses; purpose and procedures of conducting interviews and their rights to decline participation whenever inconveniences were truly felt in the process.

## Result

In-depth interviews and FGDs were held with leaders and members of *iddirs*. It was found that interviewees generally felt that they were being helped by their *iddirs*. For example, though their *iddir* doesn’t provide financial assistance, one of the *iddir’s* leaders noted: Long time served leader,

*Our iddir, beyond its major objectives, has been helping its members. For instance, if one of the members gets sick, gives birth or gets married, we contribute money and it will be given in cash; or else materials such as baby clothes will be bought for members who gave birth. Moreover, since we live in the same neighborhood we share each other’s materials.*

During a discussion by one of the focus groups, a participant explained that (Relatively a young woman, FGD,)

*“We have got more social benefits than financial ones from our iddir”.*

The participants also frequently spoke of “mutual support”. As one young woman during FGD stated that:

*“Since I joined this iddir, I realized how members help each other. They offer emotional help through expression of sympathy. I am very happy with that”.*

Furthermore, a currently retired leader who served for long time noted:

*“Despite its very minimal capital, our iddir helps members in many ways. For example, during holidays we contribute as much money as we can to enable financially weak members celebrate happily. Furthermore, we usually visit sick members. We also help them financially”.*

In addition, most participants of the discussions and the interviewees expressed that they feel a sense of caring environment in their *iddirs* which they could not find elsewhere. A relatively young woman briefly reported

*“Members’ concern and compassion often inspire me. That is why I keep participating actively”.*

Besides, all the interviewees and focus group discussions participants emphasized the importance of setting up credit facilities in their *iddirs*. However, they mentioned the inability of their respective *iddirs* in assisting members with their financial needs. One major difficulty mentioned was lack of capital. Chairperson of *Yemichael iddir*, one of the discussant among the *iddirs* under discussion, pointed out that

*“Our members know that credit facilities are important. Equally important, they know that they couldn’t contribute a big sum of money as they live on subsistence. But we believe that whatever small the contribution maybe it has paramount importance. As the chairperson put it in her own words, “And kedada yidefinal” which literally means, it solves at least one problem”.*

Additionally, a remark by a member interviewee clearly indicates how much her fellow members are interested to set up credit facility; yet unable to do so. A widow, members’ summarized the situation as,

*“Many of us want to have a group based saving and credit system so as to support ourselves economically. But, I do not think this dream would come true, mainly due to financial problem”.*

Like the interviewees, the FGDs’ participants were quick to point out lack of capital as the main constraint. A middle aged woman during FGD stated that

*“…almost all of the members are poor. This made it difficult for us to have strong financial capacity and establish saving and credit schemes”.*

Another participant in a relatively young woman, members’ added

*“If we had had the institutional credit scheme, we would have benefited more”.*

The FGDs’ participants had reservation regarding the number of members who could lend them money. However, a senior respondent recalled how members used to provide money to each other in times of needs. But an older woman, members’ understands why they are unable to do it now:

*“In old times, death among members or their relatives was not as alarming as it is today. Hence, our monthly contribution used to be saved. But now we are getting poorer and poorer while the number of deaths is increasing alarmingly. We ended up failing even to contribute the regular due”.*

With regard to members’ interest to borrow money from banks, one elderly woman of FGDs’ participant sadly reported that

*“We wish to borrow money from banks but we have not been able to do so, largely for lack of collateral and out of fear of the interest rate”*.

In women’s *iddirs* the indirect support members may get is also worth mentioning. For instance, an interviewed member explained her experience as:

*“I have a lot of problems. I sometimes consider myself unlucky and feel hopeless. Do you know why? I am a widow, jobless and mother of five. The family is financially dependent on two of my sons, who are shoe shiners. I used to bake injera for hotels. One day, while I was in the kitchen baking injera I collapsed. I was then rushed to a local health center which later sent me to a government hospital. The doctors told me that I have anemia. I became bedridden…. One thing I need to tell you is that I couldn’t afford the price for medication. However, I was fortunate that I had a very sympathetic fellow member whose relative owns a pharmacy. She told him all my problems, and he provided me the prescribed tablets for free”.*

The in-depth interviews further demonstrated that the women perceived that the contribution of iddir in their well-being was more indispensable than the contributions of other voluntary associations they ever know, such as *iqub* and *mahiber*. Especially *iddir* was regarded as crucial and unique in meeting emotional needs. For example, when a member interviewee was asked why she joined the *iddir* she participates in, she responded

*“One of the major reasons that I joined this iddir is because I want to feel emotionally secured. Joining other associations cannot bring me such security”.*

Similarly, a chairperson of *Yemichael Iddir* leaders also noted that

*“Women’s iddir has a great deal of contribution to the members than other similar associations, even more than men’s iddir, especially in terms of information sharing and condolence. For example, our iddir by-law necessitates members’ presence for three consecutive days during fellow members’ grief. But if you look at men’s iddir, their presence lasts usually about a day. Moreover, in case of iqub, for instance, the very concern for members is the money, not the social ties”.*

Recently, NGOs have gained critical lessons that *iddirs* are essential components in the implementation of their programs. However, a retired chairperson complained,

*“I heard from my relatives in Dire Dawa that their iddir works with NGOs, and they are happy with that. However, I have never heard of such a kind of partnership in our case”.*

Moreover, this study found that *iddirs’* meetings are ideal places where women share experience; discuss issues of pressing concern and their worries. As one of the FGDs participants noted,

*“When we meet we discuss almost everything, such as health issues of our families, market related issues, even in some instances we openly discuss about our husbands’ misbehavior”.*

The other participant further elaborated

*“Our meetings help us to bring issues previously thought of as private into public sphere. This in turn gives us a chance to come together, exchange ideas and forge relationships”.*

Also emphasizing the importance of experience sharing, an interviewee said,

*“Older women have a great deal of experience which they can share with us. It is useful because they can show us the safest methods to deal with issues and the possible problems we may face along the way. It also conveys truthfulness, for it is offered by people who themselves went through”.*

When asked what their *iddirs* did in helping members apart from their traditional objectives, one elderly woman FGD’s participant noted:

“*There was a poor helpless woman with six children who was beaten regularly by an errant alcoholic husband who earns more than 600 birr (1 USD = 14 birr) a month. However, he was not willing to provide her money for household expenditure. Finally, after she did let us know her problem we advised her to take the case into court and the court decided that half of his salary to be payable to her. More or less she is okay now. Usually she even advises others to share their problems*”.

During the in-depth interviews with members one woman similarly noted:

*“This iddir has helped me out a lot. It is really a place where fellow members offer you practical assistance during emergencies.… Especially for someone who is poor like me the caring reaction is very crucial”.*

Furthermore, she described how she benefited from the mutual support in her *iddir*:

*“Eight years ago it was a bad year for me. My husband was sick for two consecutive years. I was financially dependent on him. When he became sick I had double burden: looking after him and working as a daily laborer in a construction company on a daily basis. But I had to stop when his health was getting worse; because there was no one to look after him regularly. Nonetheless, I lost him. Really I was hopeless. I had nothing to do. I had no one to talk to. Life became completely dark to me. I always used to cry. I felt like the most unlucky person on earth. I even could have been a lot worse if I had not found help from the fellow members. I found the emotionally supportive environment very useful. When I had lost all hope they did more than I deserve”.*

What is more, a number of results have been identified from the FGDs and the interviews with key informants, but the following emerged as particularly important themes.

### Opportunities for social interaction, risk sharing and development of friendships

There is widespread consensus concerning the importance of social networks and social interaction to quality of life; because absence of meaningful relationships and lack of social contacts are typical risk factors for stress. Most participants, however, reported a high level of satisfaction with their social networks as they develop risk sharing. This in turn clearly prevents the accumulation of grievances. As an older woman interviewed member said,

*“When my only son died I went crazy. I mean I didn’t know what to do and what to hope for, but fellow members were typically sources of emotional as well as instrumental support. They were there for me then. They became over compassionate. I think if I didn’t have fellow members’ supports I don’t know what I would be”.*

Conversely, a minority of participants were less interested that the social network sometimes is misused for gossiping. For instance, one interviewee remarked that:

*“It might be assumed that all members do have friendly relationships, and advises would be received with great warmth. However, there are members who enjoy rumor. In short, all members are not positive thinkers. Thus, they create disharmony among members. For example, if you mistakenly did something wrong, you might hear some members talking behind your back as if you did it purposely. This affects your self-esteem and the way others treat you”.*

### Dispute resolution

Ethiopians have informal means of dispute resolution in which individuals discuss and resolve differences with the help of a third party, usually elder persons. As expressed by interviewees, this is also true in their *iddirs*. An older woman members’ interview succinctly put it

*“When arguments do occur every member tries to resolve it quickly. Of course, the friendship and solidarity among members permit faster dispute resolution and prevent feeling of resentments”.*

### Sharing and using timely information more effectively

Access to information and sharing it appropriately were reported to be important for members’ lives. Especially, petty trading being the main field of economic activity for majority of women in the area, reliable information about how, where and when to buy and sell goods and services is vital. The following quotation taken from an interviewed member illustrates it more clearly:

*“I am a petty trader. I buy and sell spices in Merkato. Recently my profitability has positively been influenced by the information I gained from a fellow member who lives in a house next to mine. She frequently travels to north Shoa to buy butter. Looking at how she was benefiting I asked her if she was willing to search for me any feasible market in north Shoa that I could make profits better than here. Afterward she searched and recommended me to buy garlic from there and sell it in Merkato. I never used to go out of Addis for trading purpose. Thanks to that lady; I am making profits”.*

Besides, when another member was asked “What impact does access to information have on your family members?” she replied,

*“It is very useful. But being subject to subordination, women’s idea is often neglected. For example, my husband is not comfortable with any information that I want to share with him. Because he usually says that any information I share with fellow members is ‘wishful thinking’”.*

### Lower level of funeral services anxiety

Many participants expressed that funeral service is really a concern and also frustrating. These arise mostly because society attaches high values to the funeral service. Above all, members value the fact that by participating in *iddir* their families’ body would be buried properly. Such belief inevitably avoids their frustration. One FGD participant noted

*“Iddir is a resource in case of emergency. For instance, for burial service I never lean on my relatives who live in other places. Instead I rely on my iddir and neighbors; because as the saying goes ‘Better a neighbor than a relative who lives far-away’”.*

### Improvement of self confidence and leadership role

Participation helped them to develop their self confidence to discuss in public on a range of issues, such as marriage, poverty and in some instance even politics. One of the young house wife members interviewed concisely noted

*“My participation helped me develop confidence to express my ideas. I used to be very shy, let alone in public, even with my families. Relatively speaking, I can now express my ideas, at least to my closest friends”.*

Another petty trader members’ interviewee similarly stated,

*“At the beginning, I was scared in the group meetings because of the difference in age between me and other fellow members. But after a while, I felt as if they were my age group; because they were friendly”.*

In the same way, a story of another woman further illustrates how members’ self-confidence grows over time:

*“When I joined this iddir, I couldn’t express my ideas and what I desire. One day a friend of mine and member was not able to come to a meeting place and she told me to tell her reasons to members for being absent on her behalf. I couldn’t say ‘No’ because she was my best friend, and at the same time I was afraid of speaking in front of all members. I finally told to a member who sat next to me and she told the other members. But I can now talk in front of all members without fear, if I need to. I will never at all feel nervous. You know, I had never thought I could do this”.*

The self-confidence that members developed also galvanized them to leadership roles. As one *Yemichael Iddir’s* chairperson, leaders’ remarked:

*“Earlier members were unwilling to take up leadership roles. Some of the prominent reasons for their unwillingness were lack of confidence, fear of being challenged and lack of education. Hence, members used to prefer to have the same leaders re-elected. However, except some members, most of them are currently willing to be leaders”.*

Usually women leaders of informal institutions do not establish relationships with government officials but the aforementioned interviewee added

*“The leadership role I held brought me into official networks. For instance, I was represented by my iddir members at public events”.*

### Reciprocity and coexistence

Human beings are distinctly vulnerable to a range of problems; some can be solved individually and yet many others may require cooperation. When people feel that they will one day face similar problems that other faced, reciprocity tends to arise naturally. Consequently, in *iddir* because of the inevitability of death of members or their families, members are guided by a principle of balanced reciprocity. For instance, one interviewee reported,

*“I always try to help fellow members because they regularly visited and assisted me when I lost my beloved mother. They helped me combat hopelessness”.*

Besides, the participatory structure of *iddir* and the way it built solidarity creates mutual interdependence and mutual coexistence amongst members. An interviewed young house wife, members’ noted

*“The fact that you are rich doesn’t help you manage all your troubles. Like it or not, there is no way that you can afford to live isolated”.*

### Trust

Mutual trust among members was reported as the bedrock of their relationships. A growing body of research suggests that where trust flourishes, day-to-day interactions will be facilitated. As a rough rule of thumb, people also collaborate with persons who are trustworthy. One interviewee spoke about how trust lubricates cooperation among members,

*“Since trust creates a social obligation you strive for a common good, knowing that others will also do so. This is because what ties the group together is the trust among its members not rules. You simply trust them to act as expected”.*

Women view their participation in iddir as a choice forever. One interviewee noted,

*“Traditionally, women’s groupings are regarded in an unambiguously negative light. Any group discussion is taken as gossiping. Undeniably, many men characterize the behavior of women as talkative for nothing. As a result, they consider all kinds of gatherings this way. However, for sure, women are altruistic. They want to have good relationships with their neighbors and acquaintances. That is why they actively participate in every social activity. You know, they don’t want their family to be detached. On the other hand, men are somewhat careless to social ties”.*

Women’s iddirs enhance strong personal relationships and they have extended psychological services for the deceased families. As expressed by a young house wife, members’ interviewee,

*“The fact that women are highly sensitive to grief necessitates extended emotional support by members to a grieving member”.* She further added

*“When women are in trouble they need to be with others who went through similar problems. This is because they feel hopeful when they meet women who had undergone similar experiences”.*

## Discussions

The purpose of this study is to examine the role and significance of self help groups for empowering women and promoting social transformation. In addition, the study sheds light on the general infrastructure required to incorporate in women’s iddirs for social capital and women empowerment. The results also suggested that participation in *iddirs* aided the women in many ways. Firstly, it is clear that members are from different backgrounds: different levels of education, different social and economic status. As a result, members benefited from the provision of information through their social network. For example, interviews conducted with members revealed that through their networks they gained vital information on a range of issues. As an interviewee explained, the market related information, for example, improved her profit and as a result she found her social networks valuable. This finding is in consistence with argument that information accessibility is one of the main reasons for investing into social network (Leskošek and Dragoš 2004).

For many participants, experience sharing is of utmost importance Majority of the participants also believe that involvement in such experience sharing and discussions enables them get assistances from fellow members. A research done in Cameroon likewise revealed that women were keen to engage in a range of forms of collective work to increase their access to networks and support (Mayoux 2001).

In addition, majority of FGDs’ participants stressed that their *iddirs* have served as suitable forums for expression of essential opinions, and to positively influence one another’s behavior. It is clear, therefore, that the social capital in women’s *iddirs* serves for experience sharing, discussing imperative concerns and information exchange.

Secondly and notably, women’s *iddirs* are also considered as important sources of emotional support. Our finding is supported by various literatures that show neighbor networks make a bigger contribution to resilience (Breton 2001) and people’s company both during and after funeral of a loved one contributes to psychological wellness for the bereaved family (Seifu 1968). Furthermore, the service of the members in accompanying the coffin and keeping company with bereaved have a social significance apart from their obvious therapeutic value (Gedamu F: Ethnic associations in Ethiopia and the maintenance of urban/rural relationships, with special reference to the Alemgana Walamo Road Construction Association, Unpublished) and helping other has even a therapeutic effect on the helper (Mok 2005).

Similarly, those who perceived their social network as strong felt that emotional support and acceptance were clearly important, particularly in the initial stages of HIV infection, when stigma and perceived differences were critical issues than physical debilitation (Ciambrone 2002). According to Alula and Damen (2000) *iddirs* are also among the most important institutions for the successful implementation of the multisectoral responses to HIV pandemic. Therefore, as vulnerable part of the community and as this major health problem results in immense psychological harms, women’s participation in *iddirs* can pave them the way to get emotional supports if they are affected and/or infected (Damen 2000).

Furthermore, it is obvious that involvement in group activities helps to build up members’ confidence. In addition to their overwhelming importance for emotional support, *iddirs* fulfill functions of labor services (Checkway 1997) and members willingly help each other and Sharing tasks is an essential aspect of social networks (Breton 2001). This finding is similar to our finding.

Certainly the roles of women’s *iddirs* are not limited to informational, emotional and labor support. Participation in *iddirs* can also be as much about process of strengthening solidarity, friendships and mutual help as it is about expanding social networks. It is true that sense of togetherness develops when people realize that they have a common problem (death of family members is the chief common problem in case of *iddir’s* members) and that they face difficult to manage it if they don’t cooperate.

Overall, findings of this study revealed that the social capital in women’s *iddirs* enables members to overcome emotional stress, exchange information, forge solidarity, strengthen mutual support and trust, and obtain material and labor supports.

When residents are involved in trusting, egalitarian networks, their cooperation establishes a community context that supports economic development (Duncan and Lamborghini 1994). Therefore, although the social implications of women’s *iddirs* outweigh the economic ones, in the present study an attempt has been made to analyze whether and to what extent participation in *iddirs* promotes economic well-being.

Studies on *iddirs* indicate that besides their social roles they do have economic roles. (Kebede W, Butterfield AK: Social networks and communication: evidence of social capital among poor women in Ethiopia, Unpublished), for example, broadly explained that “*Iddir* has both economic and social benefit, functioning as economic insurance at times of death and other crises”. Similarly, Abraham (2003) argued that financing bereavement is of course relieving specially for those who can’t ever do it otherwise (Abraham 2003).

There is still a support for the view that *iddirs* can extend their reach to other economic activities and influence quality of life. For instance, recently some *iddirs* are trying to widen their risk pooling arrangements to cover a wide variety of tasks. For example, recognizing the devastating effects of financial crises on the livelihoods of many who live in poverty, *iddirs* (especially men’s *iddirs*) began offering credit for members. Also, it deserves to be noted that in a country where there are no viable welfare services meant for the majority, even a penny is worth, especially to the poor. However, a small number of participants were arguing that the financial assistance generated from their *iddirs* is limited to the execution of proper funeral. Nevertheless, even though the financial support is small it must not be overlooked. Abraham (2003) carried the idea a step further, arguing that financial assistance during bereavement doesn’t mean that it helps the poor for their future life but gives them confidence about at least a proper burial (Abraham 2003). Moreover, labor supports of members during bereavements need to be considered as an important economic support as it eases considerable burden for grieving members. Similarly, Aredo (2003) pointed out that it is true that the bulk of financial assistance from *iddir* covers funeral expenses and provides support to be bereaved family members (Aredo 2003). However, in particular urban areas, *iddir* has several functions other than the often-disapproved after-death services. There are *iddirs* which provide medical insurance (such provision covers a fraction of total expense). Abraham (2003) further pointed out that “*iddirs* are used as the bank of the poor (credit scheme associations) in times of emergencies such as sickness, wedding ceremonies, renovation of houses, etc.” (Abraham 2003). This argument might be applicable to men’s *iddirs* (probably the unit of analysis in Abraham’s study was men’s *iddirs*), but not oftenest for women’s *iddirs*, at least for majority of women participated in this study. Actually there might be significant financial assistance from women’s *iddirs* in the future, as they achieve financial strength. However, the interviewees and FGDs’ participants of this study rarely mentioned their *iddirs*’ credit scheme. Indeed, it is arguable that where women are destitute how can they save money and assist others? Also there are enough cases to substantiate that women’s *iddirs* are financially weak. For example, in one study a quarter of 87 women’s *iddirs* experienced zero balance (Tigist L: Economic analysis of social network: empirical study on selected women iddir in Addis Ababa, Unpublished). Hence, it is wrong to equate economic contributions of men’s *iddirs* with that of women’s.

On the other hand, a recent study show that when credit given to women results in greater impact on household welfare than when it is given for men (Kevane and Wydick 2001). This is because women often utilize a greater amount of their earnings on their family and domestic expenses. However, while the participatory nature of *iddirs* makes them suitable for credit mechanisms, as it has been discussed earlier, majority of women would not be able to get credit from their *iddirs* mainly because of lack of capital. In addition, intimidating formality of banks (for example, automated interest payment) and the inability of women to produce collaterals forced them not to use these institutions even if they are available nearby. This is also reflected in this study. For example majority of participants hesitated to approach banks, don’t have the guts only because of their low economic status.

 (Aredo Interestingly, despite lack of finance and intimidating formality of banks, almost all participants seemed to be attracted to formulating saving and credit mechanisms in their respective iddirs. If applicable it would be benefiting to women; because iddirs do have flexible and simple rules that are easy to understand by everyone and easy to enforce sanctions by the group members. Besides, even if most of the members surveyed are poor, the more urbanized and monetized an economy, the higher is the role of cash transactions in *iddir*^2003^).

The study should be interpreted with its own Limitations. The cross-sectional design limits to make generalizations that this approach implies. Research on voluntary associations is still in its infancy. It is particularly important to bear in mind that the ways in which women use their *iddirs* to manage their day-to-day lives often remain under-researched. Though attempts were made to highlight the main characteristics of *iddir*, the emphasis in this study is very much on the role than the structure and nature of women’s *iddirs*.

## Conclusion for development

Women’s *iddirs* are the viable basis in the creation of social capital. They are viable in the sense that they create strong, active and connected communities, which in turn plays crucial roles in promoting solutions to social and economic challenges of women. Furthermore, women’s *iddirs* enabled members to have interaction, and this in turn encouraged discussion on issues related to social and economic activities. The results have shown that although there is a room in *iddirs* to serve as banks of the poor, there was a general consensus among almost the entire study participants that their *iddirs* were unable to offer financial assistances. Similarly, women were aware that they will need to borrow in the future from the same source they were very alert of the fact that they should maintain a good record with the micro finance institution (Pitamber 2003). Furthermore, the present study has clearly demonstrated interest on the part of the study participants to collaborate with the government and non-governmental organizations. With a concerted financial support, women’s *iddirs* can be platforms to set up micro-credit associations.

In light of the conclusions drawn above, a few suggestions can be made. Besides, as there is no strong documentation and registration of currently operating women’s *iddirs* in the city, government agencies need to work on these areas. To effect economic change in women’s lives, banks may provide loans without interest or with reasonable interest till the women are capable of paying the stated bank interest rate. Enabling women’s *iddirs* to be independent of borrowing from banks is also indispensable. Moreover, trainings on effective use of credits need to be arranged.

## References

[CR1] Abraham P, Pankhurst A (2003). The role of iddir in development of the city of Addis Ababa. Iddirs participation and development. Proceedings of the Ethiopian national conference, 20–21 December.

[CR2] (2007). Sub cities of Addis. Retrieved December, 2007.

[CR3] Aleme F (1973). A study of like mequas abebe sefer iddir.

[CR4] Aredo D (1993). The informal and semi-formal financial sectors in Ethiopia: a study of the iqqub, iddir and savings and credit co-operatives.

[CR5] Aredo D (1998). The iddir theory and practice.

[CR6] Aredo D, Pankhurst A (2003). Iddir: a look at a form of social capital. Iddirs participation and development. Proceedings of the Ethiopian national conference, 20–21 December.

[CR7] Aredo D, Asefa G (1998). Civil society organizations in development: indigenous institutions in the Cheha Wereda of the Gurage Zone.

[CR8] Atim C (1998). Contributions of mutual health organizations to financing delivery and access to health care: synthesis of research in nine West and Central African countries.

[CR9] Beck EL, Eichler M (2000). Consensus organizing: a practice model for community building. J Community Pract.

[CR10] Bown EA (2000). Community capacity antecedents and consequences. J Community Pract.

[CR11] Breton M (2001). Neighborhood resiliency. J Community Pract.

[CR12] Checkway B (1997). Core concepts for community change. J Community Pract.

[CR13] Ciambrone D (2002). Informal networks among women with HIV/AIDS: present support and future prospects. Qual Health Res.

[CR14] Damen PAHM (2000). The iddir in Ethiopia: historical development, and potential role in HIV/AIDS prevention and control. Northeast Afr Stud.

[CR15] Dercon S, De Weerdt J, Bold T, Pankhurst A (2006). Group-based funeral insurance in Ethiopia and Tanzania. World Dev.

[CR16] Duncan CM, Lamborghini N (1994). Poverty and social context in remote rural communities. Rural Sociol.

[CR17] Jutting JP (2003). Do community-based health insurance schemes improve poor people’s access to health care? Evidence from rural Senegal. World Dev.

[CR18] Kevane M, Wydick B (2001). Microenterpirse lending to female entrepreneurs: sacrificing economic growth for poverty allevation?. World Dev.

[CR19] Leskošek V, Dragoš S (2004). Community and social capital in Slovenia: the impact of transition. Eur J Soc Work.

[CR20] Mayoux L, Doornbos M, Saith A, White B (2001). Tackling the down side: social capital, women’s empowerment and micro-finance in Cameroon. Development and change, vol 32, issue 3.

[CR21] Mekuria B (1973). Edir: its role in development and social change in Addis Ababa.

[CR22] Mok B (2005). Self-help groups for empowerment and social change: findings and insights from an empirical study in Hong Kong. J Community Pract.

[CR23] Pankhurst A (1998). The role of indigenous associations in development, their past involvement, and potentials a comparison of burial, credit, migrant, and religious-social associations and institutions in development.

[CR24] Pitamber S (2003). Factors impeding the poverty reduction capacity of micro-credit: some field observations from Malawi and Ethiopia.

[CR25] Rooy V (1998). Civil society and the aid industry.

[CR26] Sable G (1986). Ekub edir and meredeja mahber as potential development tools in Addis Ababa. Symposium on the centenary of Addis Ababa, IES, University of Addis Ababa, 24–25 November.

[CR27] Seifu A (1968). Eder in Addis Ababa: a sociological study. Ethiop Obs.

[CR28] Sitz L (1995). Global issues: an introduction.

[CR29] Yan M (2004). Bridging the fragmented community: revitalizing settlement houses in the global era. J Community Pract.

